# Disentangled diffusion model for 3D molecular generation with protein–ligand interaction priors

**DOI:** 10.1093/bioinformatics/btag165

**Published:** 2026-05-05

**Authors:** Zhilin Huang, Ling Yang, Chujun Qin, Yifei Xing, Hongyang Yu, Xiangxin Zhou, Bing Zheng, Yu Wang, Xin Gao, Wenming Yang

**Affiliations:** Shenzhen International Graduate School, Tsinghua University, Shenzhen, 518000, China; Institute of Perceptual Intelligence, Peng Cheng Laboratory, Shenzhen, 518000, China; Institute of Medical Technology, Peking University, Beijing, 100080, China; China Southern Power Dispatching and Control Center, China Southern Power Grid, Guangzhou, 510700, China; Institute of Perceptual Intelligence, Peng Cheng Laboratory, Shenzhen, 518000, China; China Southern Power Dispatching and Control Center, China Southern Power Grid, Guangzhou, 510700, China; Institute of Computing, University of Chinese Academy of Sciences, Beijing, 100080, China; Institute of Perceptual Intelligence, Peng Cheng Laboratory, Shenzhen, 518000, China; Institute of Computing, University of Chinese Academy of Sciences, Beijing, 100080, China; School of Artificial Intelligence, University of Chinese Academy of Sciences, Beijing, 100080, China; Shenzhen International Graduate School, Tsinghua University, Shenzhen, 518000, China; Department of Network Intelligence, Pengcheng Laboratory, Shenzhen, 518000, China; Department of Network Intelligence, Pengcheng Laboratory, Shenzhen, 518000, China; Computer Science Program, Computer, Electrical and Mathematical Sciences and Engineering Division, King Abdullah University of Science and Technology (KAUST), Thuwal, 23955-6900, Kingdom of Saudi Arabia; Center of Excellence for Smart Health (KCSH), King Abdullah University of Science and Technology (KAUST), Thuwal, 23955-6900, Kingdom of Saudi Arabia; Center of Excellence on Generative AI, King Abdullah University of Science and Technology (KAUST), Thuwal, 23955-6900, Kingdom of Saudi Arabia; Shenzhen International Graduate School, Tsinghua University, Shenzhen, 518000, China; Institute of Perceptual Intelligence, Peng Cheng Laboratory, Shenzhen, 518000, China

## Abstract

**Motivation:**

Structure-based drug design (SBDD) aims to generate ligand molecules that tightly bind to specific protein targets, a critical step in drug discovery. Diffusion models have shown promise for this task, yet existing methods struggle to effectively incorporate protein–ligand interaction priors during generation. Most approaches rely on protein-specific structural priors that remain fixed throughout generation, limiting molecular diversity and failing to capture the dynamic interplay between protein pockets and ligand atoms, which is essential for achieving high binding affinity.

**Results:**

We propose DPDiff, a disentangled prior-conditioned diffusion model for protein-specific 3D molecular generation. DPDiff introduces two complementary interaction prior networks that capture geometry-based spatial interactions and sequence-based interactions robust to structural noise. During generation, the model dynamically extracts interaction priors using intermediate diffusion predictions and adaptively fuses them via a time-dependent adapter. A disentangled denoising network balances prior guidance with generative flexibility. Experiments on the CrossDocked2020 dataset demonstrate that DPDiff generates molecules with more realistic 3D structures and state-of-the-art binding affinities, achieving an average Vina Dock score of −8.58 and a high affinity ratio of 69.4%, outperforming existing methods while maintaining favorable drug-likeness and synthetic accessibility.

**Availability and implementation:**

The source code of DPDiff is available at https://github.com/ZerinHwang03/DPDiff.

## 1 Introduction

Designing molecule ligands capable of binding to specific protein targets and subsequently altering their function, a process known as *structure-based drug design* (SBDD) (Blundell 1996, Amzel 1998, Anderson 2003, Van Montfort and Workman 2017, Batool *et al.* 2019, Jiménez-Luna *et al.* 2020, Krishnan *et al.* 2021), is a fundamental problem in drug discovery, offering the potential for substantial therapeutic advancements. SBDD requires models to synthesize drug-like molecules with stable three-dimensional configurations and strong binding capabilities to their protein targets. Nevertheless, this task is complex and demands considerable computational resources due to the enormous space of synthetically feasible chemicals (Ragoza *et al.* 2022) and freedom degree of both compound and protein structures (Hawkins 2017). Traditional computational methods (Drews 2000, Sliwoski *et al.* 2014, Leelananda and Lindert 2016, Sinha and Vohora 2018), such as virtual screening (Kitchen *et al.* 2004, McInnes 2007, Lionta *et al.* 2014), discover molecules by iteratively (i) placing molecules from existing databases into the protein pocket cavity and (ii) filtering the molecules based on criteria such as energy estimation and toxicity by experimental assays. Despite their widespread applications, these approaches suffer from two significant limitations. Firstly, naive exhaustive searches in the massive chemical space—ranging from $10^{60}$ to $100^{100}$ depending on the size of desired molecules—are extremely expensive. Secondly, this workflow is limited by existing knowledge, thus it is infeasible to explore and generate molecular structures which are not already recorded in the existing databases.

Recently, several new generative methods have been proposed for the SBDD task (Li *et al.* 2021, Luo *et al.* 2021, Peng *et al.* 2022, Powers *et al.* 2022, Ragoza *et al.* 2022, Zhang *et al.* 2023), which learn to generate ligand molecules by modeling the complex spatial and chemical interaction features of the binding site. For instance, some methods adopt autoregressive models (ARMs) (Luo and Ji 2021, Peng *et al.* 2022) and show promising results in SBDD tasks, generating 3D molecules by iteratively adding atoms or bonds based on the target binding site. However, ARMs tend to suffer from error accumulation, and it is difficult to find an optimal generation order, which is both nontrivial for 3D molecular graphs. Aiming to address these limitations of ARMs, recent works (Guan *et al.* 2023a, Schneuing *et al.* 2024, Lin *et al.* 2025) adopt diffusion models (Ho *et al.* 2020, Song *et al.* 2020, 2021, Nichol and Dhariwal 2021) to model the distribution of atom types and positions from a standard Gaussian prior with a post-processing steps to assign bonds. These diffusion-based SBDD methods develop SE(3)-equivariant diffusion models (Hoogeboom *et al.* 2022) to capture both local and global spatial interactions between atoms and have achieved more promising performance compared with previous autoregressive models. Despite achieving promising performance, it is still difficult for these diffusion-based methods to generate molecules that satisfy biological metrics such as binding affinity. This difficulty mainly arises from the extensive search space of poses within and between molecules. Moreover, generating molecules from scratch makes the generation process more challenging to optimize and may lead to suboptimal performance. To address this issue, some diffusion-based methods attempt to incorporate external protein–ligand binding prior information to compress the model’s search space for generating molecules. DecompDiff (Guan *et al.* 2023b) and IRDiff (Huang *et al.* 2024a) employ static binding priors through anchor generation or molecular retrieval mechanisms, but their reliance on external software, like AlphaSpace (Rooklin *et al.* 2015) or precomputed databases introduces operational complexity while limiting diversity. More critically, these protein-specific priors remain fixed during generation, failing to capture dynamic target-ligand interactions essential for tight binding. The subsequent IPDiff (Huang *et al.* 2024b) advances this direction by introducing geometry-aware interaction priors with dual guidance mechanisms. However, its effectiveness is hindered by a critical training-sampling discrepancy: while utilizing the protein–ligand complexes with precise structures given in the training dataset during training process, the model must rely on progressively refined but inherently noisy molecular predictions during the training process. This mismatch propagates structural inaccuracies through prior networks, exacerbating exposure bias and ultimately compromising generation quality.

To address these limitations, we propose a disentangled protein-conditioned diffusion model (**DPDiff**) for the SBDD task. First, we establish complementary interaction prior networks: the geometry-sensitive GNet captures precise spatial relationships from protein–ligand complexes, while the SNet models the interaction between proteins and molecules and learns their binding potential based on sequential information only. Therefore, SNet has the capability to avoid being misled by the noise in molecular structure and spatial coordinates. During training, both networks process authentic protein–ligand pairs but adaptively transition to using progressively refined molecular estimates during sampling. Then, a temporal prior fusion adapter dynamically balances these inputs, emphasizing SNet’s noise tolerance in early sampling stages when molecular predictions are least reliable, and then gradually incorporating GNet’s geometric precision as structures stabilize. Finally, our disentangled denoising architecture employs parallel conditional and unconditional branches that share coordinate predictions but maintain separate feature spaces, enabling prior-guided refinement without over-constraining the model’s generative capacity. We benchmark our model on the CrossDocked2020 dataset, evaluating 10 000 generated molecules in terms of molecule structure, molecular properties, and binding degree to the given protein pocket. The generated molecules can effectively bind to the provided specific protein pockets, achieving an average Vina Dock of −8.58 among 10 000 generated molecules, outperforming current SOTA methods.

## 2 Materials and methods

### 2.1 Overview of DPDiff

**Figure 1:**
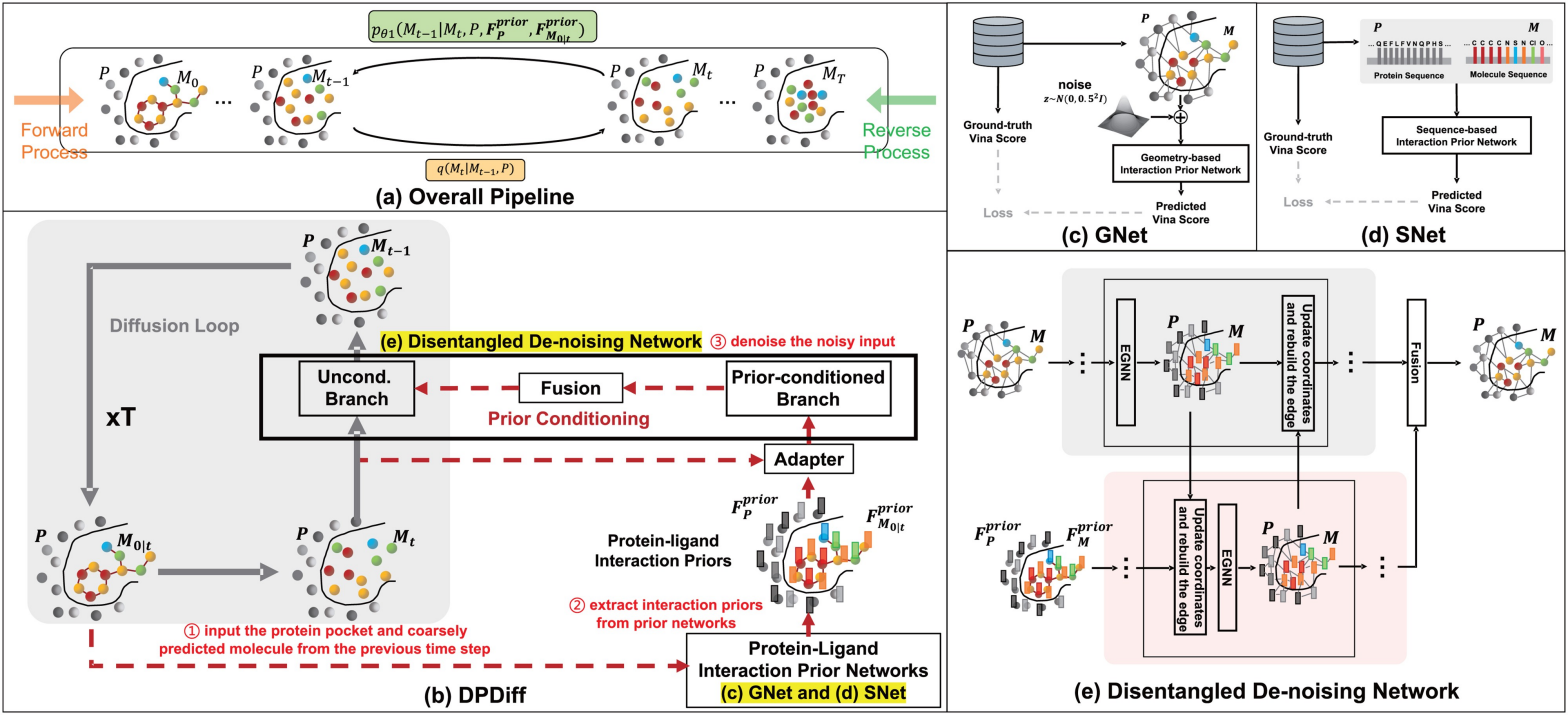
The (a) overall pipeline of DPDiff for designing protein-specific ligand molecules includes the forward and backward processes. (b) DPDiff integrates interaction priors from the pretrained protein–ligand interaction prior networks (c) GNet and (d) SNet to enhance the subsequent diffusion generation process. Then, a (e) disentangled denoising network is proposed to make better use of useful interaction hints and avoid misleading noise in the extracted interaction prior.

We propose DPDiff, a disentangled prior-conditioned diffusion framework for target-aware 3D molecule generation as shown in Fig. 1a and b. Following previous SBDD methods (Huang *et al.* 2024a), we aim to co-design both residue types (sequences) and 3D structures of the molecule ligand that can fit and bind with target protein pockets. To be specific, the target (protein) and ligand molecule can be represented as $P={\left\{(x_{\left(i\right)}^{P},v_{\left(i\right)}^{P})\right\}}_{i=1}^{N_{P}}$ and $M={\left\{(x_{\left(i\right)}^{M},v_{\left(i\right)}^{M})\right\}}_{i=1}^{N_{M}}$, respectively. Here $N_{P}$ (resp. $N_{M}$) refers to the number of atoms of the protein P (resp. the ligand molecule M). $x\in R^{3}$ and $v\in R^{K}$ denote the position and type of the atom respectively. In the sequel, matrices are denoted by uppercase boldface. For a matrix X, $x_{i}$ denotes the vector on its *i*-th row, and $X_{1:N}$ denotes the submatrix comprising its first to *N*-th rows. For brevity, the ligand molecule is denoted as $M=[X^{M},V^{M}]$ where $X^{M}\in R^{N_{M}\times 3}$ and $V^{M}\in R^{N_{M}\times K}$, and the protein is denoted as $P=[X^{P},V^{P}]$ where $X^{P}\in R^{N_{P}\times 3}$ and $V^{P}\in R^{N_{P}\times K}$.

### 2.2 Modeling protein–ligand interactions prior networks

We introduce two different interaction prior models following (Huang *et al.* 2024a) to capture the interaction priors at two different levels.

#### 2.2.1 GNet

The first interaction prior model based on protein-molecule geometric structures directly models the interaction priors by utilizing the relative positional information between atoms in proteins and molecules from the complexes, as shown in Fig. 1c. GNet consists of SE(3)-equivariant neural networks (Satorras *et al.* 2021) and cross-attention layers (Hou *et al.* 2019, Borgeaud *et al.* 2022). Two shallow fully-connected SE(3)-equivariant neural networks are applied on the graphs of the protein $G^{P}$ and ligand molecule $G^{M}$, respectively, to learn the intramolecular interactions. Another shallow fully-connected SE(3)-equivariant neural network is applied on the graphs of the complexes graph $G^{C}=G^{M}\cup G^{P}$ which is constructed by $G^{M}$ and $G^{P}$, in order to modeling the intermolecular interactions. Given the complex graph $G^{C}$, the *l*-th SE(3)-equivariant layer works as follows:


(1) $$h_{i}^{C,l+1}=h_{i}^{C,l}+\sum_{j\in N_{C}(i)}f_{h}^{C,l}\left(\left\|x_{i}^{C,l}-x_{j}^{C,l}\right\|,h_{i}^{C,l},h_{j}^{C,l}\right),$$

(2) $$x_{i}^{C,l+1}=x_{i}^{C,l}+\sum_{j\in N_{C}(i)}\left(x_{i}^{C,l}-x_{j}^{C,l}\right)f_{x}^{C,l}\left(\left\|x_{i}^{C,l}-x_{j}^{C,l}\right\|,h_{i}^{C,l+1},h_{j}^{C,l+1}\right)$$

where $h_{i}^{C,l+1}\in R^{d}$ and $x_{i}^{C,l+1}\in R^{3}$ are SE(3)-invariant and equivariant hidden states of the atom *i* of the ligand after the *l*-th SE(3)-equivariant layer, respectively. $N_{C}(i)$ stands for the set of neighbors of atom *i* on $G^{C}$, and the initial hidden state $h_{i}^{C,0}$ is obtained by an embedding layer that encodes atom information. Then, a graph attention layer is introduced to model atom-wise intra- and inter-molecular interactions of complexes graph, which essentially accounts for the binding affinity.


(3) $$f_{i}^{\mathrm{geo}}=h_{i}^{C,L}+\sum_{j\in N_{C}(i)}f_{\mathrm{GAT}}^{C}\left(\frac{h_{i}^{C,L}\cdot h_{j}^{C,L}}{\left||h_{i}^{C,L}||\cdot ||h_{j}^{C,L}|\right|},h_{i}^{C,L},h_{j}^{C,L}\right)$$

Finally, interactive representations of complexes $F^{C}=[f_{0}^{C},\ldots ,f_{N_{P}+N_{M}-1}^{C}]$ are aggregated into a global feature, and a linear layer is utilized to predict the binding affinity.

#### 2.2.2 SNet

The prior extraction during sampling is based on the coarsely predicted molecular structures from the previous time step, which is different from the training processes. Therefore, directly extracting the prior from the protein–ligand complex with relative positional relationships, inaccurate molecular structures and relative position prediction results will introduce a lot of noise into the prior easily.

To alleviate the effect of misleading noise in protein–molecule geometric structures, we introduce an interaction prior network based on the sequence information only, as shown in Fig. 1d. Compared with the sampling process based on GNet, the geometric noise introduced by the roughly predicted molecular structure in the previous time step will not affect the extraction of protein-molecule interaction priors by SNet at the current time step. Therefore, the sampling process can be kept as consistent as possible with the protein-molecule interaction prior information used in the training process, resulting in more stable generation results. Specifically, SNet first utilizes graph attention layers to independently model the protein pocket and molecular structures and then employs additional graph attention layers to further model the interaction relationships between the encoded protein pocket and molecules. Given a ligand graph $G^{M}$, the *l*-th graph attention layer works as follows:


(4) $$f_{i}^{M}=h_{i}^{M,L}+\sum_{j\in N_{M}(i)}f_{\mathrm{GAT}}^{M}\left(\frac{h_{i}^{M,L}\cdot h_{j}^{M,L}}{\left||h_{i}^{M,L}||\cdot ||h_{j}^{M,L}|\right|},h_{i}^{M,L},h_{j}^{M,L}\right)$$

where $h_{i}^{M,l+1}\in R^{d}$ are the SE(3)-invariant hidden states of the atom *i* of the ligand after the *l*-th graph attention layer, respectively. $N_{M}(i)$ stands for the set of neighbors of atom *i* on $G^{M}$, and the initial hidden state $h_{i}^{M,0}$ is obtained by an embedding layer that encodes atom sequential information. And the given protein graph $G^{P}$, $h_{i}^{P,l}$ can be derived in the same way. Then, the SE(3)-invariant features $H^{M,L}\in R^{N_{M}\times d}$ and $H^{P,L}\in R^{N_{P}\times d}$ are first concatenated along the first dimension. Consistent with GNet, another graph attention layer is introduced to model both intramolecular and intermolecular interactions of sequential protein–ligand pairs:


(5) $$\begin{array}{c} f_{i}^{\mathrm{seq}}=h_{i}^{M\cup P,L}+\\ \sum_{j\in N_{M\cup P}(i)}f_{\mathrm{GAT}}^{M\cup P}\left(\frac{h_{i}^{M\cup P,L}\cdot h_{j}^{M\cup P,L}}{\left||h_{i}^{M\cup P,L}||\cdot ||h_{j}^{M\cup P,L}|\right|},h_{i}^{M\cup P,L},h_{j}^{M\cup P,L}\right) \end{array}$$

where $H^{M\cup P}=[h_{0}^{M},\ldots ,h_{N_{M}-1}^{M},h_{0}^{P},\ldots ,h_{N_{P}-1}^{P}]$ denotes $H^{M}$ and $H^{P}$ are concatenated along the first dimension. Finally, the interactive representation $F^{M\cup P}=[f_{0}^{M\cup P},\ldots ,f_{N_{P}+N_{M}-1}^{M\cup P}]$ are atom-wisely aggregated into a global feature, and a linear layer is utilized to predict the binding affinity.

#### 2.2.3 Ensemble the priors

Finally, our method ensembles these two types of interaction priors and introduces them into the conditional branch of DPDiff through a learnable prior adapter, allowing the denoising network to adaptively allocate different attention weights to these different types of priors through the end-to-end model optimization.


(6) $$F^{\mathrm{prior}}=f_{\mathrm{adapter}}\left(F^{\mathrm{seq}},F^{\mathrm{geo}},t\right)$$

where the adapter $f_{\mathrm{adapter}}$ takes two interaction prior representations and time step *t* as input. This ensures the introduction of high-quality protein–ligand interaction priors while alleviating the impact of noise in the interaction prior extraction due to inaccurate molecule structure predictions during the sampling process.

### 2.3 Disentangled prior-conditioned diffusion model

The SBDD task from the perspective of generative models can be defined as generating ligand molecules that can bind to a given protein binding site. The target (protein) and ligand molecule can be represented as $P={\left\{(x_{i}^{P},v_{i}^{P})\right\}}_{i=1}^{N_{P}}$ and $M={\left\{(x_{i}^{M},v_{i}^{M})\right\}}_{i=1}^{N_{M}}$, respectively. Here $N_{P}$ (resp. $N_{M}$) refers to the number of atoms of the protein P (resp. the ligand molecule M). $x\in R^{3}$ and $v\in R^{K}$ denote the position and type of the atom respectively. In the sequel, matrices are denoted by uppercase boldface. For a matrix X, $x_{i}$ denotes the vector on its *i*-th row, and $X_{1:N}$ denotes the submatrix comprising its first to *N*-th rows. For brevity, the ligand molecule is denoted as $M=[X^{M},V^{M}]$ where $X^{M}\in R^{N_{M}\times 3}$ and $V^{M}\in R^{N_{M}\times K}$, and the protein is denoted as $P=[X^{P},V^{P}]$ where $X^{P}\in R^{N_{P}\times 3}$ and $V^{P}\in R^{N_{P}\times K}$. The task can be formulated as modeling the conditional distribution p(M|P). Recently, diffusion models (Ho *et al.* 2020, Song *et al.* 2021, Rombach *et al.* 2022) have achieved promising performance in SBDD tasks (Guan *et al.* 2023a, Schneuing *et al.* 2024, Lin *et al.* 2025). The types and positions of the ligand molecular atoms are modeled by DDPMs (Ho *et al.* 2020), while the number of atoms $N_{M}$ is usually sampled from an empirical distribution (Hoogeboom *et al.* 2022, Guan *et al.* 2023a) or predicted by a neural network (Lin *et al.* 2025), and the chemical bonds are generated by post-processing programs. We define $\beta_{t}$ (t=1,…,T) as fixed variance schedules, $\alpha_{t}=1-\beta_{t}$, ${\bar{\alpha }}_{t}=\prod_{s=1}^{t}\alpha_{s}$, ${\bar{\beta }}_{t}=1-{\bar{\alpha }}_{t}$.

#### 2.3.1 Reverse process

Different from the traditional diffusion models for SBDD described above, our proposed DPDiff performs the denoising process through a disentangled prior-conditioned denoising network.

In the approximated reverse process, also known as the generative process, a neural network parameterized by θ1 learns to recover data by iteratively denoising. The reverse transition kernel can be approximated with predicted atom types ${\hat{v}}_{0|t,i}^{M}$ and atom positions ${\hat{x}}_{0|t,i}^{M}$ at time step *t* as follows:


(7) $$\begin{array}{c} p_{\theta 1}(M_{t-1}|M_{t},P)=\prod_{i=1}^{N_{M}}N(x_{t-1,i}^{M};\tilde{\mu }(x_{t,i}^{M},{\hat{x}}_{0|t,i}^{M}),{\tilde{\beta }}_{t}I)\\ \cdot C(v_{t-1,i}^{M}|\tilde{c}(v_{t,i}^{M},{\hat{v}}_{0|t,i}^{M})). \end{array}$$

Unlike traditional equivariant diffusion models used for SBDD, the denoising network of DPDiff, as shown in Fig. 1e, consists of two parallel branches, the unconditional branch and the prior-conditioned branch, each composed of *l* layers of equivariant graph networks. In the unconditional branch, the input is the component of the noised molecule $M_{t}$ at time step *t* and the given condition protein pocket P, consistent with previous work. In the prior-conditioned branch, besides taking $M_{t}$ and P as inputs, we also introduce interaction prior information about the given conditional protein pocket and the target generated molecule, which is obtained using the SNet and GNet pre-trained in the first stage. Specifically, we firstly feed the given protein pocket P and ligand molecule $M_{0}$ into the pre-trained SNet and GNet and the interaction prior adapter to obtain features $F^{\mathrm{prior}}=[F^{\mathrm{prior},M},F^{\mathrm{prior},P}]$ that indicate the implicit interaction correlations between the protein pocket and the molecule ligand, where [·] indicates the concatenation along the atom dimension. It is worth noting that the molecule structure $M_{0}$ we feed into the pre-trained prior networks is the given reference molecule structures provided by the training data during the training stage, and it is the coarsely estimated molecule structure from the previous time step during the sampling denoising phase, since the real molecule structure is the generation target and is inaccessible. Subsequently, the unconditional branch directly takes the component $C_{t}=[M_{t},P]$, and time step *t* as inputs to perform the denoising process, where [·] indicates the concatenation along the atom dimension. In contrast, the prior-conditioned branch concatenates $C_{t}$, *t*, and the prior information $\left[F^{\mathrm{prior},M},F^{\mathrm{prior},P}\right]$ along the channel dimension as inputs, achieving denoising of $M_{t}$ under the guidance of prior information.

In the *l*-th layer of the equivariant graph networks for the unconditional and prior-conditioned branches, $f_{\mathrm{uncond}}^{l}(\cdot )$ and $f_{\mathrm{prior}}^{l}(\cdot )$, the inputs are the output features and coordinates, $\left(h^{\mathrm{uncond},l},x^{\mathrm{uncond},l}\right)$ and $\left(h^{\mathrm{prior},l},x^{\mathrm{prior},l}\right)$ from the (l−1)-th layer, respectively. The equivariant graph network layers in the unconditional branch take the hidden states of each atom output by the previous equivariant graph network layer as input, and use the updated coordinates of each atom from the previous equivariant graph network in the prior-conditioned branch to construct the edges for each atom, further updating the hidden states and coordinates of each atom. Similarly, in the prior-conditioned branch, the equivariant graph network layers take the hidden states of each atom output by the previous equivariant graph network layer as input, and use the updated coordinates of each atom from the equivariant graph network in the unconditional branch to construct the edges for each atom, completing the update of the hidden states and coordinates of each atom in the prior-conditioned branch. After the information propagation and updates through *L* layers, the hidden states of each atom output by the two branches are fused in a linear combination manner with certain weights to obtain the final output hidden states for each atom, while the 3D coordinates from the prior-conditioned branch are used as the final output positions of each atom in space. It is worth noting that only the coordinates are exchanged between the two branches at each layer, while the hidden states are linearly combined only in the last layer. This is to better decouple the information processing processes between the two branches and avoid the impact of noise in the prior. The entire process can be formulated as:


(8) $$h^{\mathrm{uncond},l+1},x^{\mathrm{uncond},l+1}=f_{\mathrm{uncond}}^{l}(x^{\mathrm{prior},l},h^{\mathrm{uncond},l})$$

(9) $$h^{\mathrm{prior},l+1},x^{\mathrm{prior},l+1}=f_{\mathrm{prior}}^{l}(x^{\mathrm{uncond},l+1},h^{\mathrm{prior},l})$$

(10) $$h^{L}=(1-\eta )\cdot h^{\mathrm{uncond},L}+\eta \cdot h^{\mathrm{prior},L},\;x^{L}=x^{\mathrm{prior},L}$$

where η is a predefined scalar value, we set it to 0.75 in practice.

#### 2.3.2 Forward process and the training paradigm

For the forward process, we directly utilize the ground-truth molecule for extracting interaction priors. The details of the forward process and the training paradigm of DPDiff are provided in the Supplementary Materials, available as supplementary data at *Bioinformatics* online.

## 3 Experimental results and discussion

### 3.1 Implementation details

The implementation details of the interaction prior network and DPDiff are provided in the Supplementary Material, available as supplementary data at *Bioinformatics* online.

### 3.2 Benchmarking generated molecule ligands

We comprehensively evaluate the generated molecules from three perspectives: molecular structures, target binding affinity, and molecular properties. In terms of molecular structures, we calculate the Jensen-Shannon divergences (JSD) in the empirical distributions of atom/bond distances between generated molecules and ground-truth ones provided in the test set. To estimate the target binding affinity, following previous work (Luo *et al.* 2021, Ragoza *et al.* 2022, Guan *et al.* 2023a, Huang *et al.* 2024a), we adopt AutoDock Vina (Eberhardt *et al.* 2021) to compute and report the mean and median of binding-related metrics, including Vina Score, Vina Min, Vina Dock, and High Affinity. Vina Score directly estimates the binding affinity based on generated 3D molecules; Vina Min performs a local structure minimization before estimation; Vina Dock involves an additional re-docking process and reflects the best possible binding affinity; High Affinity measures the ratio of how many generated molecules bind better than the ground-truth molecule per test protein. To evaluate molecular properties, we utilize QED, SA, Diversity as metrics following (Luo *et al.* 2021, Ragoza *et al.* 2022). QED is a simple quantitative estimation of drug-likeness combining several desirable molecular properties; SA is a measurement of the difficulty of synthesizing ligands; Diversity is computed as the average pairwise dissimilarity between all generated ligands.

We compare our model with recent representative methods for SBDD. LiGAN (Ragoza *et al.* 2022) is a conditional VAE model trained on an atomic density grid representation of protein–ligand structures. GraphBP (Liu *et al.* 2022b), AR (Luo *et al.* 2021) and Pocket2Mol (Peng *et al.* 2022) are autoregressive schemes that generate 3D molecules atoms conditioned on the protein pocket and previous generated atoms. TargetDiff (Guan *et al.* 2023a), DecompDiff (Guan *et al.* 2023b), IRDiff (Huang *et al.* 2024a) and IPDiff (Huang *et al.* 2024b) are recent state-of-the-art non-autoregressive diffusion-based SBDD models, and these methods all introduce external priors into the diffusion model to enhance the generation performance.

### 3.3 Generative capabilities of DPDiff

We evaluate the effectiveness of DPDiff by comparing with two types of SBDD methods: non-diffusion methods and diffusion-based methods, in Table 1 and Fig. 3a in the Supplementary Material. Our DPDiff significantly outperforms non-diffusion baselines in binding-related metrics. Notably, DPDiff also surpasses the strong autoregressive method Pocket2Mol by a large margin of 53.4%, 33.0%, and 27.2% in Med. Vina Score, Vina Min, and Vina Dock, respectively. Compared with the state-of-the-art diffusion-based method IRDiff, DPDiff increases the binding-related metrics Med. Vina Score, Vina Min, Vina Dock by 4.6%, 5.0%, and 2.6%. In terms of high-affinity binder, we find that on average 69.4% of the DPDiff molecules show better binding affinity than the reference molecule, which is better than other baselines. These gains demonstrate that the proposed DPDiff effectively utilizes protein–ligand interaction priors from SNet and GNet to enable the generating molecules with improved target binding affinity.

**Table 1:** Summary of different properties of ground-truth molecules in the test set and molecules generated by our model and other non-diffusion and diffusion-based baselines.^a^

Methods		Vina score (↓)		Vina min (↓)		Vina dock (↓)		High affinity (↑)		QED (↑)		SA (↑)		Diversity (↑)	
		Avg.	Med.	Avg.	Med.	Avg.	Med.	Avg.	Med.	Avg.	Med.	Avg.	Med.	Avg.	Med.
Ground truth		−6.36	−6.46	−6.71	−6.49	−7.45	−7.26	–	–	0.48	0.47	0.73	0.74	–	–
**Compare with Non-diffusion**	LiGAN	–	–	–	–	−6.33	−6.20	21.1%	11.1%	0.39	0.39	0.59	0.57	0.66	0.67
	GraphBP	–	–	–	–	−4.80	−4.70	14.2%	6.7%	0.43	0.45	0.49	0.48	**0.79**	**0.78**
	AR	−5.75	−5.64	−6.18	−5.88	−6.75	−6.62	37.9%	31.0%	0.51	0.50	0.63	0.63	0.70	0.70
	Pocket2Mol	−5.14	−4.70	−6.42	−5.82	−7.15	−6.79	48.4%	51.0%	**0.56**	**0.57**	**0.74**	**0.75**	0.69	0.71
	**DPDiff**	**−5.95**	**−7.21**	**−7.46**	**−7.74**	**−8.58**	**−8.64**	**69.4**%	**74.5**%	0.50	0.51	0.57	0.56	0.74	0.73
**Compare with diffusion**	TargetDiff	−5.47	−6.30	−6.64	−6.83	−7.80	−7.91	58.1%	59.1%	0.48	0.48	0.58	0.58	0.72	0.71
	DecompDiff	−5.67	−6.04	−7.04	−7.09	−8.39	−8.43	64.4%	71.0%	0.45	0.43	**0.61**	**0.60**	0.68	0.68
	IRDiff	−6.03	−6.89	−7.27	−7.37	−8.42	−8.42	67.4%	72.7%	**0.53**	**0.54**	0.59	0.58	0.72	0.72
	IPDiff	**−6.42**	−7.01	−7.45	−7.48	−8.57	−8.51	**69.5**%	**75.5**%	0.52	0.53	**0.61**	0.59	**0.74**	**0.73**
	**DPDiff**	−5.95	**−7.21**	**−7.46**	**−7.74**	**−8.58**	**−8.64**	69.4%	74.5%	0.50	0.51	0.57	0.56	**0.74**	**0.73**

a (↑)/(↓) denotes a larger/smaller number is better. Top 2 results are highlighted with **bold text** and underlined text, respectively.

**Figure 2:**
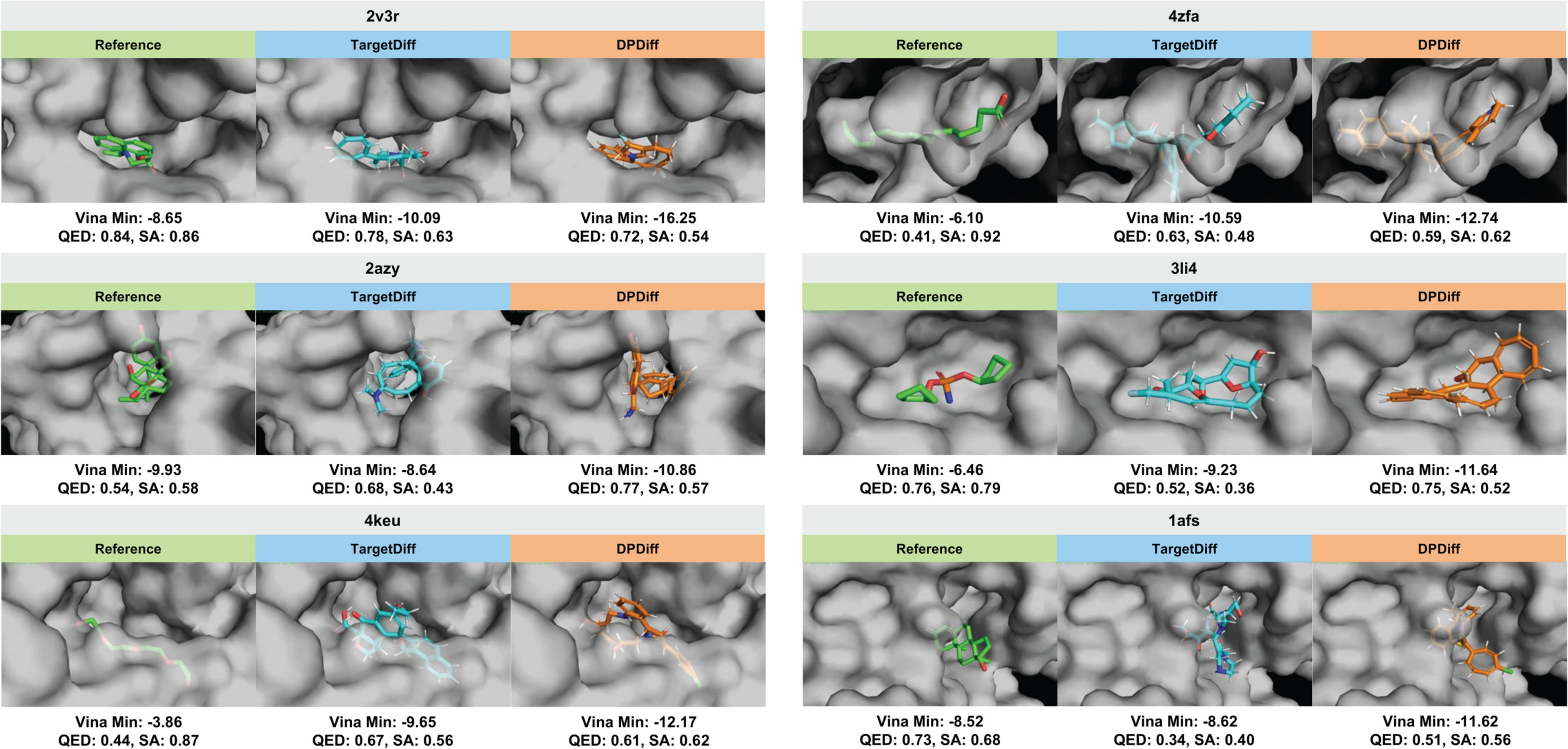
Examples of generated ligands for protein pockets (1afs, 3li4, 4zfa, 4keu, 2azy), the surface of protein pockets are visualized in white. Carbon atoms in reference ligands, ligands generated by TargetDiff (Guan et al. 2023a) and DPDiff are visualized in green, blue and orange, respectively. Vina Min, QED and SA are reported.

We present some examples of generated ligand molecules and their properties in Fig. 2. The molecules generated by our model have valid structures and reasonable binding poses to the target, which are supposed to be promising candidate ligands.

### 3.4 Effectiveness of protein–ligand interaction prior

We conducted a set of experiments to explore the impact of different interaction priors on the performance of our model. Table 2 presents the performance of our model with different types of interaction priors. Using the performance of the model without any interaction prior as a comparative baseline, we can observe that directly introducing geometry-based interaction priors (**+GNet**) degrades the model’s performance. This is because geometry-based interaction priors are pretrained with accurate protein–ligand relative positions, which makes them sensitive to the relative spatial positions of proteins and molecule ligands. During the sampling process, the interaction priors are extracted according to the inaccurately estimated spatial positions of molecules from the previous time step, introducing a significant amount of misleading noise into the extracted interaction priors. Besides, using only sequence-based interaction priors (**+SNet**) provides a very limited improvement in the model’s performance, as this interaction prior does not effectively utilize the relative spatial position relationship between proteins and molecule ligands, which is highly related to the Vina Score and Vina Min metrics. To this end, our method (**DPDiff**) simultaneously integrates these two types of interaction priors, which model the interaction priors from different perspectives, into the denoising network. Through a t-dependent interaction prior adapter, the model is able to adaptively focus on useful interaction hints in these two types of interaction prior while alleviating the effect of noise during the interference stage. We also evaluate methods by comparing the generated molecules with different number of rotatable bonds and the performance of the model equipped with different types of interaction priors at different stages of the sampling process, as presented in Fig. 4a and b in the Supplementary Material, available as supplementary data at *Bioinformatics* online.

**Table 2:** The effect of introducing different type of protein–ligand interaction priors.^a^

Methods	Vina score (↓)		Vina min (↓)		Vina dock (↓)		High affinity (↑)		QED (↑)		SA (↑)		Diversity (↑)	
	Avg.	Med.	Avg.	Med.	Avg.	Med.	Avg.	Med.	Avg.	Med.	Avg.	Med.	Avg.	Med.
baseline	−5.04	−5.75	−6.38	−6.52	−7.55	−7.72	54.2%	54.1%	0.46	0.46	0.57	0.57	0.71	0.69
+GNet	−3.58	−5.48	−5.56	−6.07	−7.15	−7.27	52.3%	52.5%	0.39	0.38	0.57	0.56	**0.76**	**0.74**
+SNet	−6.02	−6.60	−7.21	−7.19	−8.23	−8.16	62.9%	64.8%	**0.51**	**0.51**	**0.58**	**0.58**	**0.76**	**0.74**
**DPDiff**	**−6.47**	**−7.26**	**−7.64**	**−7.71**	**−8.58**	**−8.46**	**68.4**%	**72.4**%	**0.52**	**0.53**	0.57	0.57	0.74	0.71

a (↑)/(↓) denotes a larger/smaller number is better. Top 2 results are highlighted with **bold text** and underlined text, respectively.

## 4 Conclusion

Due to the vast search space, introducing external prior knowledge into generative models can effectively narrow down the search range and generate high-quality results. However, during the generation process, using the interaction priors between the target molecule structure and the protein pocket to guide the generation of molecules is a ‘chicken-or-egg’ problem since the target molecule structures are unknown before their creation. In this paper, we propose DPDiff, which fully leverages the characteristics of intermediate results in the iterative sampling of diffusion models, and gradually introduces the interaction priors between protein pockets and molecule structures during the generation process of target molecules. To avoid the model being affected by inaccurate intermediate molecule structures when extracting interaction priors, we adopt two strategies: one is to introduce interaction priors with different sensitivities to the molecule structures, and introduce a prior integration adapter, allowing the adapter to adaptively focus on useful information in the interaction priors while alleviating the misleading noise through the end-to-end optimization process of the model. On the other hand, we introduce a disentangled prior-conditioned denoising network that alternately performs generation processes guided by interaction priors and without interaction priors through two parallel branch networks, effectively incorporating interaction priors into the model while avoiding the model to over rely on external interaction priors. Extensive experiments across benchmarks demonstrate the capabilities of DPDiff generating molecules that tightly bind to the given protein pockets and have reasonable structures.

## Supplementary Material

btag165_Supplementary_Data

## Data Availability

The data underlying this article are available in the GitHub repository of TargetDiff, at https://github.com/guanjq/targetdiff. The datasets were derived from sources in the public domain: TargetDiff's GitHub repository, at https://github.com/guanjq/targetdiff#data.
