# Supplementary material for: Disentangled diffusion model for 3D molecular generation with protein–ligand interaction priors
Source: Bioinformatics. 2026 May 5;42(6):btag165. doi: 10.1093/bioinformatics/btag165 (PMC13273430; doi:10.1093/bioinformatics/btag165)
Supplement: btag165_Supplementary_Data [file btag165_supplementary_data.pdf]

Supplementary

Pesudo Code

Training Process. We summarize the training procedure of DPDiff in Algorithm 1.

Algorithm 1 Training Procedure of DPDiff

Require: Protein-ligand binding dataset $\{\mathcal{P}, \mathcal{M}\}_{i=1}^{N}$, denoising neural network ϕ_θ and interaction prior adapter ϕ_ω, pre-trained SNet and GNet
while ϕ_θ and ϕ_ω not converge **do**
  Sample diffusion time $t \in \mathcal{U}(0, \ldots, T)$
  Move the complex to make CoM of protein atoms zero
  Perturb $[\mathbf{X}_0, \mathbf{V}_0]$ to obtain $[\mathbf{X}_t, \mathbf{V}_t]$
  Embed $\mathbf{V}_t$ into $\mathbf{H}_M^0$, and embed $\mathbf{V}_P$ into $\mathbf{H}_P^0$
  Obtain different types of interaction prior features $\mathbf{F}^{seq}$ and $\mathbf{F}^{geo}$ with the pretrained SNet and GNet, respectively, based on $[\mathbf{X}_0, \mathbf{V}_0]$
  Obtain the fused interaction prior features $\mathbf{F}^{prior,\mathcal{M}}$, $\mathbf{F}^{prior,\mathcal{P}}$ by highlighting the useful information in two types of interaction prior features through the interaction prior adapter ϕ_ω
  Predict $[\hat{\mathbf{X}}_0, \hat{\mathbf{V}}_0]$ from $[\mathbf{X}_t, \mathbf{H}_M, \mathbf{F}^{prior,\mathcal{M}}]$ and $[\mathbf{X}_P, \mathbf{H}_P, \mathbf{F}^{prior,\mathcal{P}}]$
  Compute loss L with $[\hat{\mathbf{X}}_0, \hat{\mathbf{V}}_0]$ and $[\mathbf{X}_M, \mathbf{V}_M]$
  Update θ and ω by minimizing L
end while

Sampling Process. Given a protein $\mathcal{P}$, the molecules can be sampled as Algorithm 2.

Algorithm 2 Sampling Procedure of DPDiff

Require: The protein binding site $\mathcal{P}$, the learned denoising network ϕ_θ, the learned interaction prior adapter ϕ_ω, pre-trained SNet and GNet
Ensure: Generated ligand molecule $\mathcal{M}$ that binds to the protein pocket $\mathcal{P}$
Sample the number of atoms N_M of the ligand molecule $\mathcal{M}$
Move CoM of protein atoms to zero
Sample initial ligand atom coordinates $\mathbf{x}_T$ and atom types $\mathbf{v}_T$
Let $\mathbf{M}^* := [\mathbf{0}, \mathbf{0}]$
Embed $\mathbf{V}_P$ into $\mathbf{H}_P$
for t in $T, T-1, \ldots, 1$ **do**
  Embed $\mathbf{V}_t$ into $\mathbf{H}_M$
  Obtain $\mathbf{F}^{seq}$ and $\mathbf{F}^{geo}$ from the predicted $[\hat{\mathbf{X}}_0, \hat{\mathbf{V}}_0]$ at previous time step
  Obtain $\mathbf{F}^{prior,\mathcal{M}}$, $\mathbf{F}^{prior,\mathcal{P}}$ by fusing $\mathbf{F}^{seq}$ and $\mathbf{F}^{geo}$ through the adapter ϕ_ω
  Predict $[\hat{\mathbf{X}}_0, \hat{\mathbf{V}}_0]$ from $[\mathbf{X}_t, \mathbf{H}_M, \mathbf{F}^{prior,\mathcal{M}}]$ and $[\mathbf{X}_P, \mathbf{H}_P, \mathbf{F}^{prior,\mathcal{P}}]$
  Sample $\mathbf{X}_{t-1}, \mathbf{V}_{t-1}$ from the posterior p_θ
  Let $\mathbf{M}^* := [\hat{\mathbf{X}}_0, \hat{\mathbf{V}}_0]$
end for

Architecture of Protein-ligand Interaction Prior Networks

The details of the architecture of protein-ligand interaction prior networks are present in Tab. 3.

Details of Baseline Methods

LiGAN. LiGAN (Ragoza *et al.*, 2022) is the first method for generating the 3D structure of molecule ligands based on the structure of a given protein receptor. Specifically, LiGAN employs a conditional variational autoencoder trained on atomic density grid representations of docked protein-ligand structures, and constructs valid molecular conformations from the generated atomic densities through atom and bond inference procedures. Experimental results demonstrate that LiGAN can generate molecules with higher binding affinity and appropriate drug properties.

GraphBP. GraphBP (Liu *et al.*, 2022b) is an auto-regressive model that generates 3D molecules by placing atoms of specific types and locations into given protein binding sites step by step. In each step, it first uses a 3D graph neural network to obtain geometric-aware and chemically informative representations from the intermediate context, which includes the given binding site and atoms placed in previous steps. Secondly, to maintain the desired equivariance property, a local reference atom is selected according to the designed auxiliary classifiers, and a local spherical coordinate system is constructed. Finally, the type of the new atom and its relative position with respect to the constructed local coordinate system are generated via a flow model. Additionally, the method also considers generating variables sequentially to capture the underlying dependencies among them.

AR. AR (Luo *et al.*, 2021) is a novel auto-regressive model capable of generating molecular structures that bind to specific protein binding sites. Specifically, AR first uses a context encoder to learn informative representations of each atom through graph neural networks, then aggregates the representations of contextual atoms through a spatial classifier and predicts the probability of a query position being occupied by an atom of a specific chemical element. Additionally, AR includes a frontier network to determine when to stop the sampling process. With this auto-regressive sampling strategy, the model can generate valid and diverse molecules.

Pocket2Mol. Pocket2Mol (Peng *et al.*, 2022) is an auto-regressive model consisting of two main modules: one is an E(3)-equivariant graph neural network designed to capture the spatial and bonding relationships of atoms within protein pockets, and the other is a new efficient algorithm capable of conditionally sampling new drug candidates from a tractable distribution without relying on MCMC (Markov Chain Monte Carlo) methods. This network utilizes vector-based neurons and geometric vector perceptrons as fundamental blocks, jointly predicting frontier atoms, atomic positions, atom types, and chemical bonds, and performs molecular sampling in an auto-regressive manner.

TargetDiff. TargetDiff (Guan *et al.*, 2023a) is one of the pioneer end-to-end methods utilizing diffusion models to address structure-based drug design (SBDD) task. Generally, TargetDiff is a 3D equivariant diffusion model that learns a joint generative process of continuous atom coordinates and discrete atom types with an SE(3)-equivariant network, explicitly modeling the interactions between atoms in 3D space. However, TargetDiff does not consider incorporating external knowledge to assist in the generation process of small molecules. In contrast, our approach takes TargetDiff as the base model and effectively introduces external priors into the model's generative process.

DecompDiff. DecompDiff (Guan *et al.*, 2023b) is a novel diffusion model designed for SBDD tasks. By decomposing the ligand generation process into arms and scaffold, and leveraging external software AlphaSpace (Rooklin *et al.*, 2015) to obtain protein-specific information about the ligand's arms and scaffold, it completes the generation of atoms within the ligand based on these two pieces of information. Through this method, DecompDiff aims to generate molecules that are structurally reasonable and have high affinity for the target protein. However, the prior information obtained by DecompDiff through AlphaSpace is only related to the protein pocket, leading to lower diversity in the generated results. In contrast, the protein pocket and small molecule interaction priors used in our method are not only related to the protein pocket itself but also to the structure of the target molecule ligand to be generated, which helps our model generate a higher diversity of molecule structures.

Table 3. Architecture details of both SNet and GNet

Network	Module	Backbone	Input Dimensions	Output Dimensions	Blocks
SNet	Ligand Encoder	Graph Attention Layer	$N_M \times 128$	$N_M \times 128$	2
	Protein Encoder	Graph Attention Layer	$N_P \times 128$	$N_P \times 128$	2
	Interaction Layer	Graph Attention Layer	$(N_M + N_P) \times 128$	$(N_M + N_P) \times 128$	1
	Prediction Head	MLP	1×128	1×1	1
GNet	Complex Encoder	EGNN	$(N_M + N_P) \times 128$	$(N_M + N_P) \times 128$	2
	Interaction Layer	Graph Attention Layer	$(N_M + N_P) \times 128$	$(N_M + N_P) \times 128$	1
	Pooling	Sum-pooling	$(N_M + N_P) \times 128$	1×128	1
	Prediction Head	MLP	1×128	1×1	1

IRDiff. IRDiff (Huang *et al.*, 2024a), our previous method, is an innovative interaction-based retrieval-augmented 3D molecular diffusion model designed for the generation of 3D molecules that bind to specific protein targets. IRDiff incorporates a geometric protein-molecule interaction network, pre-trained with binding affinity signals, which serves as a retriever capable of estimating the binding affinity from a given protein-ligand pair. Specifically, IRDiff first retrieves target-aware ligand molecules with high binding affinity from a predefined set of reference ligands to serve as templates. Then, IRDiff extracts protein-ligand interaction priors from these ligand templates and the given protein structures to effectively enhance the molecule ligand generation process.

IPDiff. IPDiff (Huang *et al.*, 2024b), our previous method, is an innovative interaction-prior guided 3D molecular diffusion model designed for generating target-aware molecules with enhanced binding affinity. IPDiff introduces geometric protein-ligand interaction priors into both diffusion and sampling processes through a pre-trained geometry-based interaction network IPNet supervised by binding affinity signals. Specifically, IPDiff first employs IPNET to model structural and chemical interactions between protein pockets and ligand molecules. Then, it leverages these learned interaction patterns to adapt molecular diffusion trajectories through prior-shifting that injects protein-aware perturbations in the forward process, and enhance binding-aware generation through prior-conditioning that refines denoising steps using estimated complex structures in the reverse process.

The Forward Process and Training Paradigm

Forward process. In the forward diffusion process, a small Gaussian noise is gradually injected into data as a Markov chain. Because noises are only added on ligand molecules but not proteins in the diffusion process, we denote the atom positions and types of the ligand molecule at time step t as $\mathbf{X}_t^{\mathcal{M}}$ and $\mathbf{V}_t^{\mathcal{M}}$. The diffusion transition kernel can be defined as follows:

$$q(\mathbf{M}_t|\mathbf{M}_{t-1}, \mathbf{P}) = \prod_{i=1}^{N_M} \mathcal{N}(\mathbf{x}_{t,i}^{\mathcal{M}}; \sqrt{1-\beta_t}\mathbf{x}_{t-1,i}^{\mathcal{M}}, \beta_t \boldsymbol{I}) \cdot \mathcal{C}(\mathbf{v}_{t,i}^{\mathcal{M}}|(1-\beta_t)\mathbf{v}_{t-1,i}^{\mathcal{M}} + \beta_t/K), \tag{11}$$

$$q(\mathbf{M}_t|\mathbf{M}_0, \mathbf{P}) = \prod_{i=1}^{N_M} \mathcal{N}(\mathbf{x}_{i,0}^{\mathcal{M}}; \sqrt{1-\bar{\beta}_t}\mathbf{x}_{i,0}^{\mathcal{M}}, \bar{\beta}_t \boldsymbol{I}) \cdot \mathcal{C}(\mathbf{v}_{i,0}^{\mathcal{M}}|(1-\bar{\beta}_t)\mathbf{v}_{i,0}^{\mathcal{M}} + \bar{\beta}_t/K), \tag{12}$$

where $\mathcal{N}$ and $\mathcal{C}$ stand for the Gaussian and categorical distribution respectively, β_t is defined by fixed variance schedules. The corresponding posterior can be analytically derived as follows:

$$q(\mathbf{M}_{t-1}|\mathbf{M}_t, \mathbf{M}_0, \mathbf{P}) = \prod_{i=1}^{N_M} \mathcal{N}(\mathbf{x}_{t-1,i}^{\mathcal{M}}; \tilde{\mu}(\mathbf{x}_{t,i}^{\mathcal{M}}, \mathbf{x}_{0,i}^{\mathcal{M}}), \tilde{\beta}_t \boldsymbol{I}) \cdot \mathcal{C}(\mathbf{v}_{t-1,i}^{\mathcal{M}}|\tilde{c}(\mathbf{v}_{t,i}^{\mathcal{M}}, \mathbf{v}_{0,i}^{\mathcal{M}})), \tag{13}$$

where $\tilde{\mu}(\mathbf{x}_{t,i}^{\mathcal{M}}, \mathbf{x}_{0,i}^{\mathcal{M}}) = \frac{\sqrt{\bar{\alpha}_{t-1}}\beta_t}{1-\bar{\alpha}_t}\mathbf{x}_{0,i}^{\mathcal{M}} + \frac{\sqrt{\alpha_t}(1-\bar{\alpha}_{t-1})}{1-\bar{\alpha}_t}\mathbf{x}_{t,i}^{\mathcal{M}}$, $\tilde{\beta}_t = \frac{1-\bar{\alpha}_{t-1}}{1-\bar{\alpha}_t}\beta_t$, $\alpha_t = 1-\beta_t$, $\bar{\alpha}_t = \prod_{s=1}^t \alpha_s$, $\tilde{c}(\mathbf{v}_{t,i}^{\mathcal{M}}, \mathbf{v}_{0,i}^{\mathcal{M}}) = \frac{c^*}{\sum_{k=1}^K c_k^*}$, and $c^*(\mathbf{v}_{t,i}^{\mathcal{M}}, \mathbf{v}_{0,i}^{\mathcal{M}}) = [\alpha_t \mathbf{v}_{t,i}^{\mathcal{M}} + (1-\alpha_t)/K] \odot [\bar{\alpha}_{t-1}\mathbf{v}_{0,i}^{\mathcal{M}} + (1-\bar{\alpha}_{t-1})/K]$.

Training Paradigm. In the process of de novo generation of molecule structures based on given protein pockets in DPDiff, the diffusion model iteratively uses the coarse estimate of the molecular structure from the previous time step as input, together with the given protein pocket, to extract protein-molecule interaction priors from the pre-trained interaction prior network. The ideal training process would be to simulate the reverse denoising process at time step t to obtain a coarse-grained estimated molecular structure from the previous time step, and obtain the corresponding protein-molecule interactions priors for subsequent model guidance. However, the reverse sampling process of the diffusion model is a Markov process, which means for training at time step t, we need to perform an additional $T - t$ iterations to obtain the coarse-grained molecular structure predicted at step $t + 1$. This characteristic would greatly increase the model training cost. Therefore, we directly use the structure of the real reference molecule from the training dataset as input to extract the real interaction priors. Meanwhile, to simulate as much as possible the noise in the prior information brought about by inaccurate estimation of the molecule structure during the molecule sampling stage, we introduce a certain amount of perturbation to the position of each atom of the real reference molecule structure fed into the pre-trained interaction prior network during the training process, and this perturbation is related to the time step t, with the perturbation increasing as we get closer to T.

To train DPDiff (*i.e.*, optimize the evidence lower bound induced by DPDiff), we use the same objective function as (Guan *et al.*, 2023a). The atom position loss and atom type loss at time step $t-1$ are defined as follows respectively:

$$L_{t-1}^{(x)} = \frac{1}{2\tilde{\beta}_t^2}\sum_{i=1}^{N_M} \|\tilde{\mu}(\mathbf{x}_{i,t}, \mathbf{x}_{i,0}) - \tilde{\mu}(\mathbf{x}_{i,t}, \hat{\mathbf{x}}_{i,0})\|^2 = \gamma_t \sum_{i=1}^{N_M} \|\mathbf{x}_{i,0} - \hat{\mathbf{x}}_{i,0}\|; \tag{14}$$

$$L_{t-1}^{(v)} = \sum_{i=1}^{N_M}\sum_{k=1}^{K} \tilde{c}(\mathbf{v}_{i,t}, \mathbf{v}_{i,0})_k \log \frac{\tilde{c}(\mathbf{v}_{i,t}, \mathbf{v}_{i,0})_k}{\tilde{c}(\mathbf{v}_{i,t}, \hat{\mathbf{v}}_{i,0})_k}; \tag{15}$$

where $\hat{\mathbf{X}}_0$ and $\hat{\mathbf{V}}_0$ are predicted from $\mathbf{X}_t$ and $\mathbf{V}_t$, and $\gamma_t = \frac{\bar{\alpha}_{t-1}\beta_t^2}{2\tilde{\beta}_t^2(1-\bar{\alpha}_t)^2}$. Kindly recall that $\mathbf{x}_{i,t}$, $\mathbf{v}_{i,t}$, $\hat{\mathbf{x}}_{i,0}$, and $\hat{\mathbf{v}}_{i,0}$ correspond to the i-th row of $\mathbf{X}_t$, $\mathbf{V}_t$, $\hat{\mathbf{X}}_0$, and $\hat{\mathbf{V}}_0$, respectively. The final loss combines the above two losses with a hyperparameter λ as: $L = L_{t-1}^{(x)} + \lambda L_{t-1}^{(v)}$.

Implementation Details of Interaction Prior Network.

For building the protein-ligand interaction prior network SNet and GNet, we utilize the binding affinity prediction task to build up the optimization

Table 4. The effect of disentangle branch. (↑) / (↓) denotes a larger / smaller number is better. Top 2 results are highlighted with bold text and underlined text, respectively.

Methods	Vina Score (↓)		Vina Min (↓)		Vina Dock (↓)		High Affinity (↑)		QED (↑)		SA (↑)		Diversity (↑)	
	Avg.	Med.	Avg.	Med.	Avg.	Med.	Avg.	Med.	Avg.	Med.	Avg.	Med.	Avg.	Med.
baseline	-5.04	-5.75	-6.38	-6.52	-7.55	-7.72	54.2%	54.1%	0.46	0.46	0.57	0.57	0.71	0.69
classifier-free guidance	-5.80	-6.55	-6.87	-6.94	-8.18	-8.23	63.6%	65.9%	0.49	0.50	**0.59**	**0.58**	**0.75**	**0.73**
**DPDiff**	**-5.95**	**-7.21**	**-7.46**	**-7.74**	**-8.58**	**-8.64**	**69.4%**	**74.5%**	**0.50**	**0.51**	0.57	0.56	0.74	**0.73**

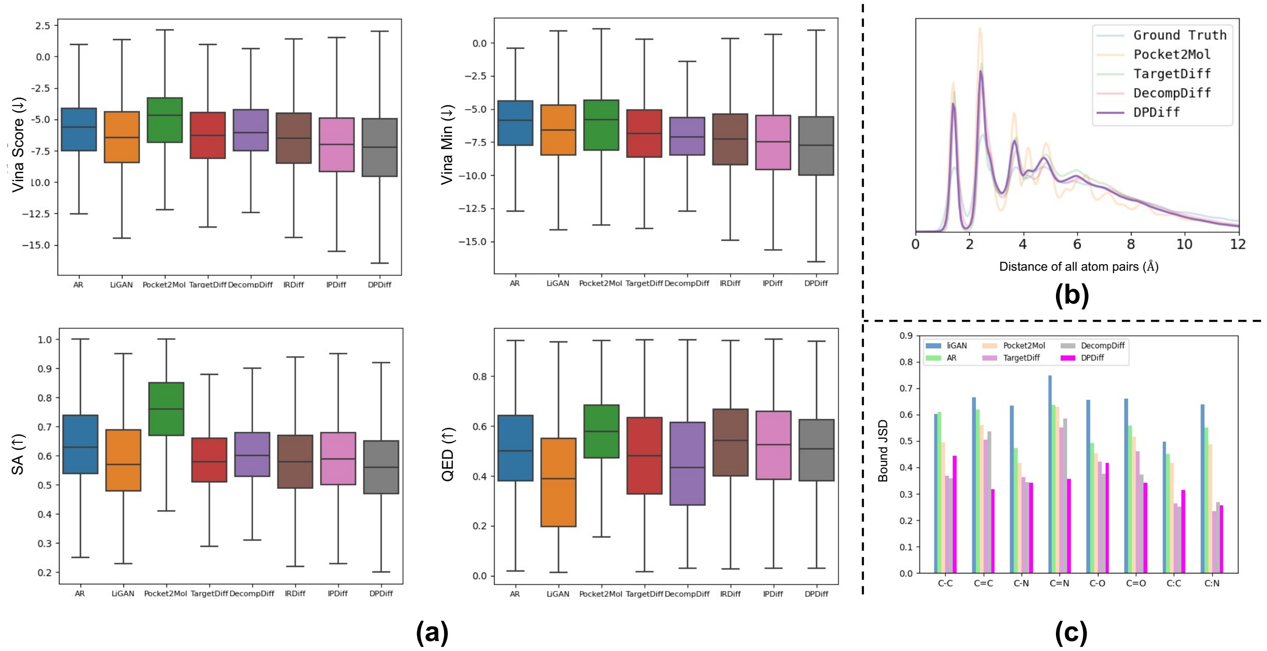

Fig. 3: Benchmarking DPDiff on CrossDocked dataset. (a), We report the benchmarking results including Vina Score, Vina Min. performance using AutoVina (Rooklin *et al.*, 2015) software, Quantitative Estimate of Drug (QED) and Synthetic Accessibility (SA). The sample size in the plots are 10000 for each model. In each box plot, the minimum value of the dataset is indicated at the lower whisker's end. The lower boundary of the box is defined by the first quartile (Q1), which is the 25th percentile. A line within the box signifies the median, or the 50th percentile, showing the data's midpoint. The upper boundary of the box is the third quartile (Q3), representing the 75th percentile. The maximum value of the dataset is marked at the end of the upper whisker. (b), We compare Jensen-Shannon divergence (JSD) between all-atom distance distributions of reference molecules in the test set and model-generated molecules. (c), We compare Jensen-Shannon divergence (JSD) between bond distance distributions of the reference molecules and the generated molecules, and lower values indicate better performances. "-", "=", and ":" represent single, double, and aromatic bonds, respectively.

objective. Following (Huang *et al.*, 2024a), to pretrain both SNet and GNet with binding affinity signals, we use the PDBbind v2016 dataset (Liu *et al.*, 2015), which is most frequently used in binding-affinity prediction tasks. Specifically, 3767 complexes are selected as training set, and the other 290 complexes are selected as testing set. For select the ideal the pre-trained protein-ligand interaction prior network, we use the benchmark on the binding affinity prediction tasks. Following (Li *et al.*, 2021), we select Root Mean Square Error (RMSE), Mean Absolute Error (MAE), Pearson's correlation coefficient (R) and the standard deviation (SD) in regression to measure the prediction error. Meanwhile, we use these metrics to select the pretrained SNet and GNet utilized in DPDiff because we believe that the ability to predict the binding affinity is highly related to the interaction modeling.

Implementation Details of DPDiff

Following (Guan *et al.*, 2023a), we use a one-hot element indicator {H, C, N, O, S, Se} and one-hot amino acid type indicator (20 types) to represent each protein atom. Similarly, each ligand atom are represented with a one-hot element indicator {C, N, O, F, P, S, Cl}. And an additional one-dimensional flag indicating whether the atoms belong to the protein or ligand are introduced. Two 1-layer MLPs are used to map the input protein and ligand into 128-dim latent spaces respectively.

Generative Capabilities of DPDiff

We also compare the molecular structures of molecules generated by our DPDiff and the other representative methods. The all-atom pairwise distance distribution of the generated molecules are plotted in Figure 3 (b). And Figure 3 (c) presents the bond distributions of the molecules generated by different methods compared against the corresponding reference empirical distributions. And our DPDiff achieves superior performance on major bond types compared to all other methods, which demonstrating the ability of DPDiff in generating stable molecular structures.

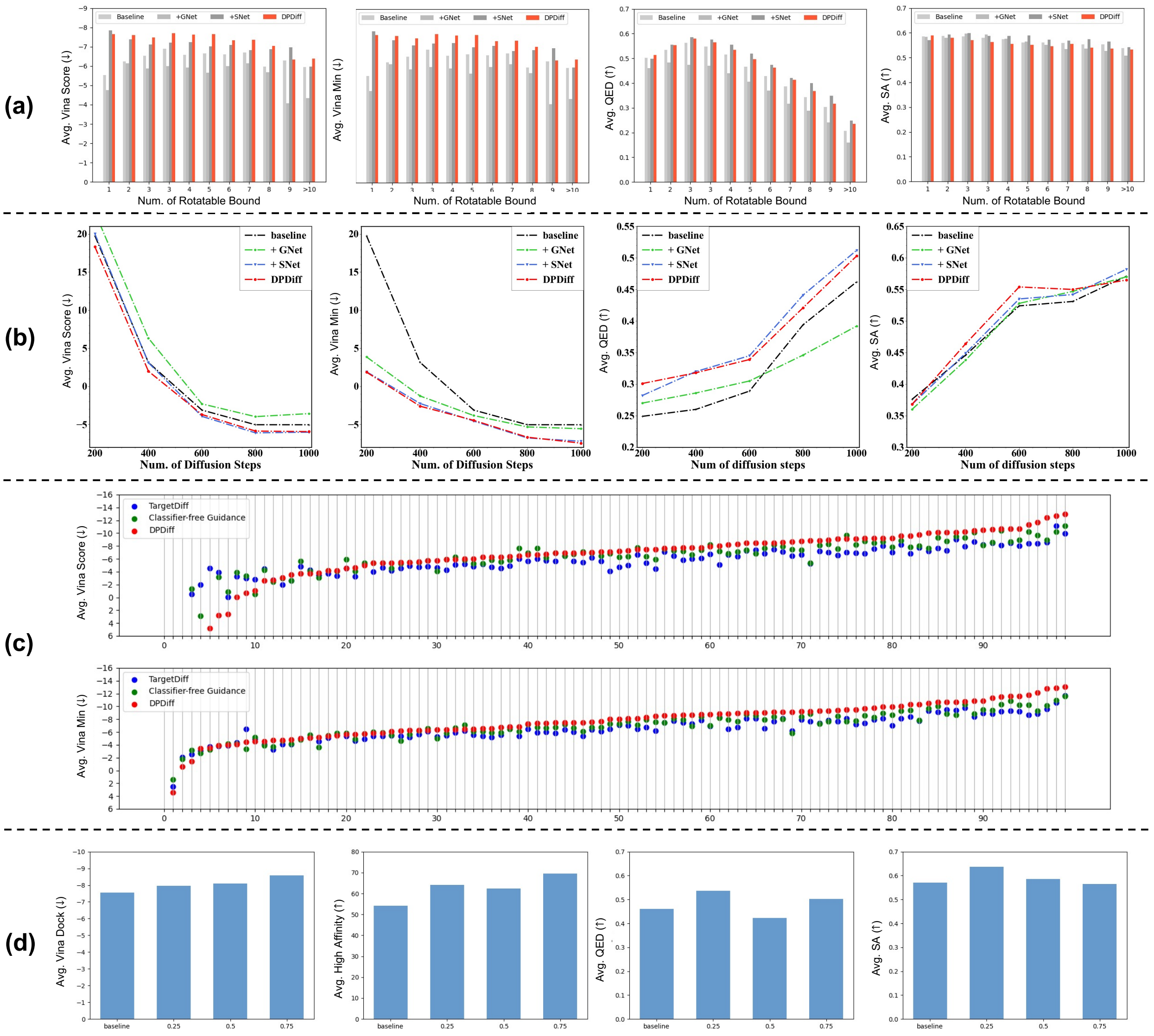

Fig. 4: To exploring the effectiveness of incorporating interaction prior into DPDiff, we incorporate different interaction priors into the baseline model and verify their performance in terms of molecular properties (QED, SA) and protein binding (Vina Score, Vina Min.). The average performance of the generated molecules with the different number of rotatable bonds is shown in (a). The average performance of the generated molecules sampled with different {200, 400, 600, 800, 1000} diffusion steps is shown in (b). To exploring the effectiveness of disentangled prior-conditioned denoising network in DPDiff, we compare it with the classifier-free guidance technique (Chen *et al.*, 2023) and verify their performance in terms of protein binding (Vina Score, Vina Min.). The average performance of the generated molecules on each of the 100 pockets in the test set is shown in (c). We also explore the effectiveness of the hyper-parameter, the fusion weight for fusing the output of the conditional and unconditional branches in our disentangled prior-conditioned denoising network, and verify their performance in terms of molecular properties and protein-molecule binding. The average performance of applying different fusion weights {0.25, 0.5, 0.75} for fusing the output of both conditional and unconditional branches is shown in (d).

Effectiveness of Protein-ligand Interaction Prior

We also evaluate methods by comparing the generated molecules with the different number of rotatable bonds as presented in Figure 4 (a). We can observe that introducing interaction priors into the base model can effectively enhance performance, even for edge cases that molecules with high number of rotatable bonds. In addition, in Figure 4 (b), we present the performance of the model equipped with different types of interaction priors at different stages of the sampling process. It can be observed that solely introducing interaction priors from SNet can help the model to achieve better performance in almost all sampling steps. Building on this, incorporating prior interaction priors with richer protein-molecule interaction priors from GNet can further enhance the model's performance at each sampling step. However, solely introducing interaction priors from GNet will lead to worse performance of the base model in terms of Vina Min. and QED metrics. This observation aligns with our design rationale: the decoupling of SNet and GNet directly addresses exposure bias arising from the training-sampling discrepancy. During training, interaction priors are extracted from ground-truth structures, whereas sampling relies on noisy intermediate predictions from previous timestep. In early stage, GNet amplifies geometric noise, propagating errors into subsequent denoising. In

contrast, SNet, which prioritizes sequence-based constraints, remains stable under noise. However, SNet solely applying SNet cannot achieve optimal results, as it ignores spatial relationships between the protein and molecule, failing to model precise binding patterns or provide effective geometric guidance. Thus, we propose a time-dependent interaction prior adapter to dynamically balance their contributions. Specifically, in early diffusion steps (high noise), the adapter prioritizes SNet to suppress geometric instability, while in later steps (low noise), it gradually increases GNet 's contribution to optimize spatial alignment. By decoupling and adaptively fusing these priors, DPDiff leverages GNet 's geometric sensitivity to generate high-affinity conformations while maintaining stability through SNet 's sequence-driven constraints.

4.1 Incorporating the Interaction Priors into the Diffusion Model

Tab. 4 and Figure 4 (c) illustrates the comparison between our disentangled prior-conditioned denoising network and the traditional denoising network equipped with the classifier-free guidance which is a technique to incorporate the conditional information into the diffusion models. Specifically, the classifier-free guidance introduces interaction priors as conditional information with a certain probability during the training process and only linearly blending the conditional and unconditional output results at the output layer during inference process. In contrast, our method introduces two parallel branches, the conditional and unconditional branches, to construct a decoupled denoising network, and applies the interaction prior to the conditional branch throughout the entire training process. By layer-wisely updating the spatial position coordinates of each graph node in both branches, it implicitly guides the update of graph node features and achieves information propagation between the two branches, thereby better integrating the interaction prior into the model's denoising process. Besides, since the disentangled prior-conditioned denoising network is designed for simultaneously de-nosing the noised structure and sequence information, the graph node features from both branches are linearly fused in the output layers with a certain fusion weights. Figure 4 (d) show the impact of different fusion weights on the final model performance. As demonstrated in Figure 4 (d), increasing the fusion weight enhances the contribution of the conditional branch, leading to improved performance on protein-ligand binding metrics such as Vina Dock. However, this comes at a trade-off with molecular property metrics like SA. At a fusion weight of 0.75, the model achieves optimal performance on binding-related metrics while maintaining competitive performance on molecular properties. Based on these empirical observations, we select 0.75 as the fusion weight in practices, prioritizing binding performance without significantly compromising molecular properties.

Complexity Analysis

We evaluate the computational complexity of TargetDiff, DecompDiff, IPDiff and DPDiff by calculating the average consuming time of sampling per 100 molecules, and present results in Tab. 5.

Table 5. Computational complexity.

Methods	TargetDiff	DecompDiff	IPDiff	DPDiff
Inference Time (s/100 mols)	1987	3218	3063	5307

More Evaluation Results

We evaluate our DPDiff and baseline methods (generate 10 ligands for each pocket provided in the test set) on physical interactions metrics through PoseCheck (Harris *et al.*, 2023), and present results in Tab. 6. Three evaluation metrics are reported. We evaluate our DPDiff and the baselines using PoseCheck (Harris *et al.*, 2023), generating 10 ligands per testset pocket. Three physical interaction-related metrics are reported in Tab. 6. (1) Avg. Clashes, the number of steric clashes between ligand and protein atoms, lower values indicate better physical plausibility. (2) Avg. VdWContact, the number of van der Waals contacts, higher values suggest tighter packing. (3) Avg. Hydrophobic, the number of hydrophobic interactions, higher values indicate better hydrophobic matching. As shown in Tab. 6, DPDiff consistently outperforms baselines across all three metrics. Fewer steric clashes, demonstrating improved physical feasibility. More van der Waals contacts and substantially more hydrophobic interactions, indicating better shape complementarity and enhanced hydrophobic matching with the binding pocket. These consistent improvements demonstrate that DPDiff generates molecules with more rational binding modes and tighter pocket integration, confirming its effectiveness over baseline methods.

Table 6. Evaluation results on PoseCheck. (↑) / (↓) denotes a larger / smaller number is better. Top 2 results are highlighted with bold text and <u>underlined text</u>, respectively.

Methods	TargetDiff	IPDiff	DPDiff
Avg. Clashes (↓)	15.62	<u>14.66</u>	**14.20**
Avg. VdWContact (↑)	<u>9.99</u>	9.97	**10.43**
Avg. Hydrophobic (↑)	5.89	<u>7.39</u>	**8.49**

Limitation

One limitation of the current DPDiff framework is its assumption of rigid protein pockets, inherited from the static structures in training datasets like CrossDocked. While this simplification aligns with common practices in structure-based drug design, it overlooks the dynamic nature of protein-ligand interactions, such as induced-fit effects. Nevertheless, DPDiff 's architecture inherently supports flexible pocket modeling. The interaction prior networks (SNet and GNet) already process protein-ligand spatial relationships as inputs, and the time-step-aware fusion adapter could be extended to handle conformational noise—for example, by adaptively suppressing geometric sensitivity to pocket flexibility in early diffusion steps. Future work will explore training on flexible pocket data (e.g., molecular dynamics trajectories) to capture conformation-dependent interaction patterns. Additionally, we plan to investigate joint denoising of ligand structures and pocket conformations, inspired by recent advances in flexible protein-ligand diffusion [1,2], to implicitly model induced-fit mechanisms. Addressing the exacerbated exposure bias from dynamic conformation sampling will require novel strategies, such as conformation-aware prior networks or hierarchical denoising schedules. These extensions aim to bridge the gap between static computational models and the dynamic reality of biological systems.

Future Work

DPDiff provides a framework that can effectively introduce external prior knowledge into the model's generation process, which creates several fruitful directions for future work. One direction is exploiting a way to introduce some molecular property-related priors when building interaction prior networks, thereby guiding the model to generate molecules with specific properties. Another direction is that the current method requires retraining the entire model when using different interaction priors, developing a zero-shot method for incorporating interaction priors into the generation model or solely fine-tuning the interaction prior adapter would greatly enhance the flexibility of the model in application.
